# Growth performance and nutrient intake of Gir x Holstein dairy calves reared in a tropical outdoor system and inoculated with rumen-derived fungi

**DOI:** 10.1007/s11250-026-05248-7

**Published:** 2026-07-21

**Authors:** Ellen Batista Pereira, Luciana Castro Geraseev, Felipe Gomes da Silva, Leilla Magalhães Queiróz, Flávio Emanuel Gomes Silva, Júlia de Melo Viana, Idael Matheus Góes Lopes, Eduardo Robson Duarte

**Affiliations:** 1 Institute of Agricultural Sciences, Federal University of Minas Gerais (UFMG), Montes Claros, MG Brazil; 2 Department of Animal Science, Federal University of Minas Gerais (UFMG), Belo Horizonte, Minas Gerais Brazil

**Keywords:** Dairy calves, Cellulolytic fungi, Probiotic supplementation, Structural growth, Semi-arid environment

## Abstract

The effects of oral inoculation with a probiotic containing *Rhodotorula mucilaginosa* and *Trichoderma longibrachiatum* were evaluated during the first 60 d of life in Gir x Holstein dairy calves across two seasons. Twenty calves were assessed in northern Minas Gerais, Brazil (*n* = 10 in period 1, late winter–spring; *n* = 10 in period 2, late summer–autumn). The study used a 2 × 2 factorial arrangement with probiotic supplementation (with or without fungal inoculation) and experimental periods as factors. Growth performance, structural development, and nutrient intake were evaluated. Fungal supplementation increased total hip width gain (4.5 vs. 3.6 cm) and tended to increase hay dry matter intake during the preweaning phase in both periods. However, supplementation did not influencied other performance parameters (*p* > 0.05). Calves evaluated during Period 2 showed greater average daily gain (0.674 vs. 0.537 kg/d) and improved feed efficiency (1.44 vs. 1.69) compared with Period 1. In conclusion, fungal inoculation enhanced structural growth, specifically hip width gain, whereas overall performance was more strongly influenced by season. Dairy calves reared under a tropical outdoor system in a semi-arid region exhibited improved growth performance during late summer and autumn.

**Supplementary Information:**

The online version contains supplementary material available at https://doi.org/10.1007/s11250-026-05248-7.

## Introduction

Calf rearing is one of the most critical phases of dairy production systems, as these animals constitute the future replacement herd and directly determine the availability of productive and healthy cows (Azevedo et al. 2014; Silva et al. 2025). Therefore, the adoption of appropriate nutritional and health management practices, together with adequate welfare conditions, is essential to promote optimal health and growth during early life (Campos et al. 2023; Oliveira et al. 2023; Silva et al. 2025).

Proper care during the first months of life is particularly important because this period is marked by profound physiological and anatomical changes in the digestive tract of ruminants (Azevedo et al. 2014). During this stage, the animal development is influenced by diet composition, feed nutritional quality and by interactions between the diet and ruminal microorganisms (Stivari et al. 2014; Santos et al. 2021). Additionally, young calves are more susceptible to diarrhea, respiratory diseases, tick-borne diseases, umbilical infections, and often experience high mortality rates (Carroll et al. 2025; Fluck et al. 2024).

Probiotic supplementation can improve calf development during early life (Carroll et al. 2025). However, many microorganisms used in these bioproducts are not native to the digestive tract of ruminants. Previous studies evaluating probiotics in crossbred calves have not reported consistent effects on body measurements (Bittar et al. 2016), suggesting that responses in structural development may depend on microbial strain, developmental stage, and environmental conditions.

Rumen-derived fungi have gained increasing attention as potential microbial additives in ruminant nutrition due to their remarkable ability to degrade fibrous forages (Abrão et al. 2014; Duarte et al. 2025). These microorganisms produce enzymes with strong fibrolytic activity (Duarte et al. 2021, 2024), which can increase energy and protein availability, stimulate the growth of ruminal microbiota, and consequently enhance animal productivity (Duarte et al. 2025; Santos et al. 2021; Stivari et al. 2014).

Duarte et al. (2023), when evaluating weaned Nellore calves (~ 8 months old), supplemented or not with a mixture of *Trichoderma longibrachiatum* and *Aspergillus terreus*, reported improved feed efficiency in supplemented animals. Similarly, Magaço et al. (2020) observed a tendency toward greater final body weight and average daily gain in weaned lambs inoculated with a *T. longibrachiatum* isolate obtained from the ovine digestive tract. In another study, supplementation with the yeast *Rhodotorula mucilaginosa*, also isolated from the digestive tract of sheep, increased dry matter intake in weaned lambs (Júnior et al. 2022). However, the use of these fungi as dietary supplements for dairy calves during the milk-feeding phase has not yet been investigated.

Therefore, microbial additives based on autochthonous fungal strains isolated from cattle raised under tropical conditions may represent a promising strategy. Such additives may contribute to the development of effective alternative products for dairy calf rearing, with potentially fewer side effects compared with conventional pharmaceutical interventions (Duarte et al. 2021). Therefore, the objective in this study was to evaluate growth performance, development, and nutrient intake of Gir x Holstein dairy calves during the suckling phase, supplemented or not with fungi isolated from the bovine digestive tract.

## Materials and methods

### Experimental site and climatic conditions

The study was conducted on a commercial dairy farm located in Bocaiúva, Minas Gerais, Brazil (16°57′00″ S, 43°42′18″ W; 992 m altitude). According to the Köppen classification, the regional climate is tropical humid with a dry winter (Alvares et al. 2014).

The experiment was a completely randomized design in a 2 × 2 factorial arrangement, consisting of fungal supplementation (with or without fungi) and two seasonal periods. Period 1 was conducted from August to December 2022 (late winter and spring), with average rainfall of 75 mm, mean temperature of 24.5 °C, and relative humidity of 49.3%. Period 2 occurred from March to July 2023 (late summer and early autumn), with average rainfall of 114 mm, mean temperature of 23.1 °C, and relative humidity of 54.7% (Suplementary Material S1) (INMET, 2023).

### Fungal strains and inoculum preparation

**Fig. 1:**
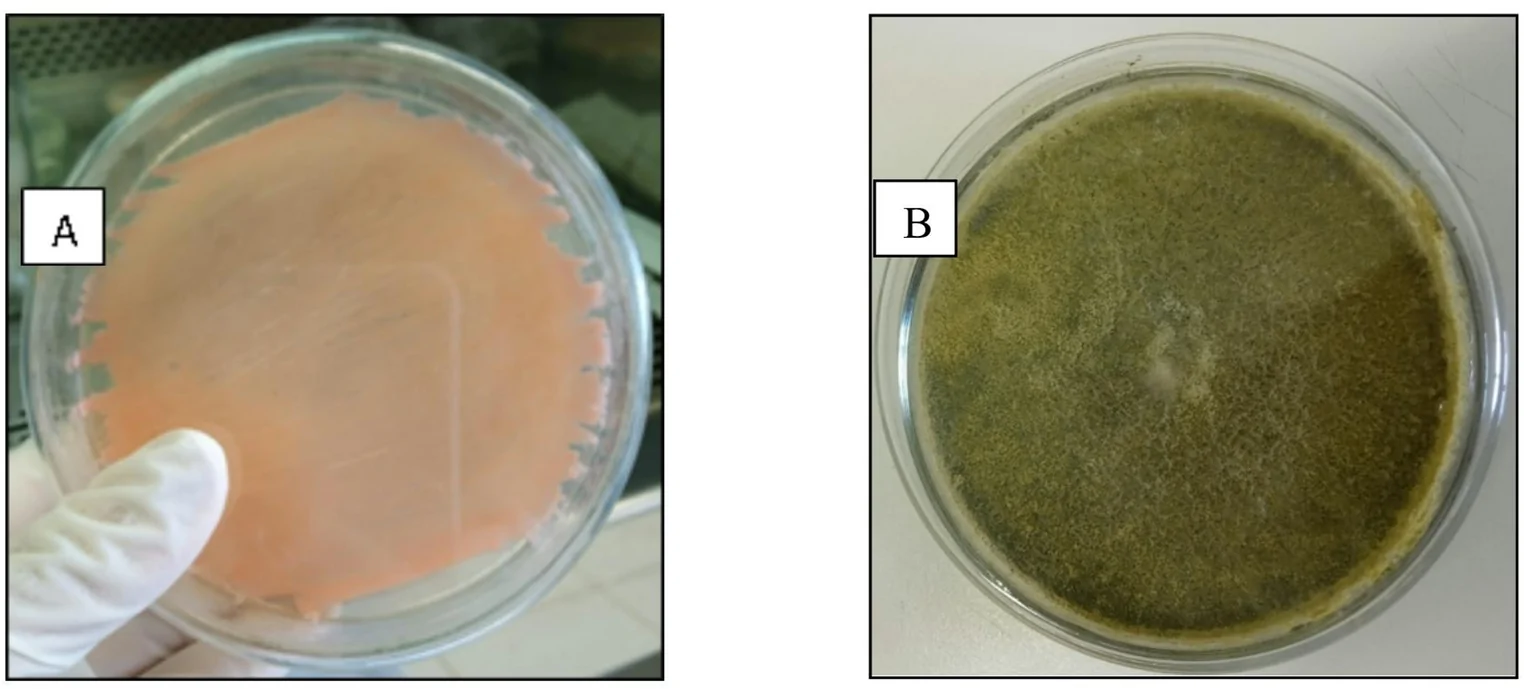
Isolates of *Rhodotorula mucilaginosa* (**A**) and *Trichoderma longibrachiatum* (**B**)

Two autochthonous ruminal fungal microorganisms were used: the yeast *Rhodotorula mucilaginosa* V16S and the filamentous fungus *Trichoderma longibrachiatum* VN20 (Fig. 1). Both isolates were obtained from the ruminal fluid of Nellore cattle grazing tropical pastures and were selected based on their high ruminal population density (> 10⁶ CFU/mL) and their ability to enhance the degradation of lignified forages (Abrão et al. 2014; Duarte et al. 2021, 2024, 2025).

Yeast identification was performed by sequencing the D1/D2 domains of the 26 S rRNA gene, whereas the filamentous fungus was identified by micromorphology and ITS region sequencing. Sequences were compared with GenBank entries using BLASTn, confirming the isolates as *R. mucilaginosa* (accession MN075224.1) and *T. longibrachiatum* (accession KF781535).

For inoculum preparation, the filamentous fungus and yeast were cultured in Sabouraud broth for seven and three days, respectively. Cultures were quantified by colony-forming unit counts and standardized to 1 × 10⁸ CFU/mL. Inocula were stored at 4 °C until use.

### Animals, housing, and neonatal management

**Fig. 2:**
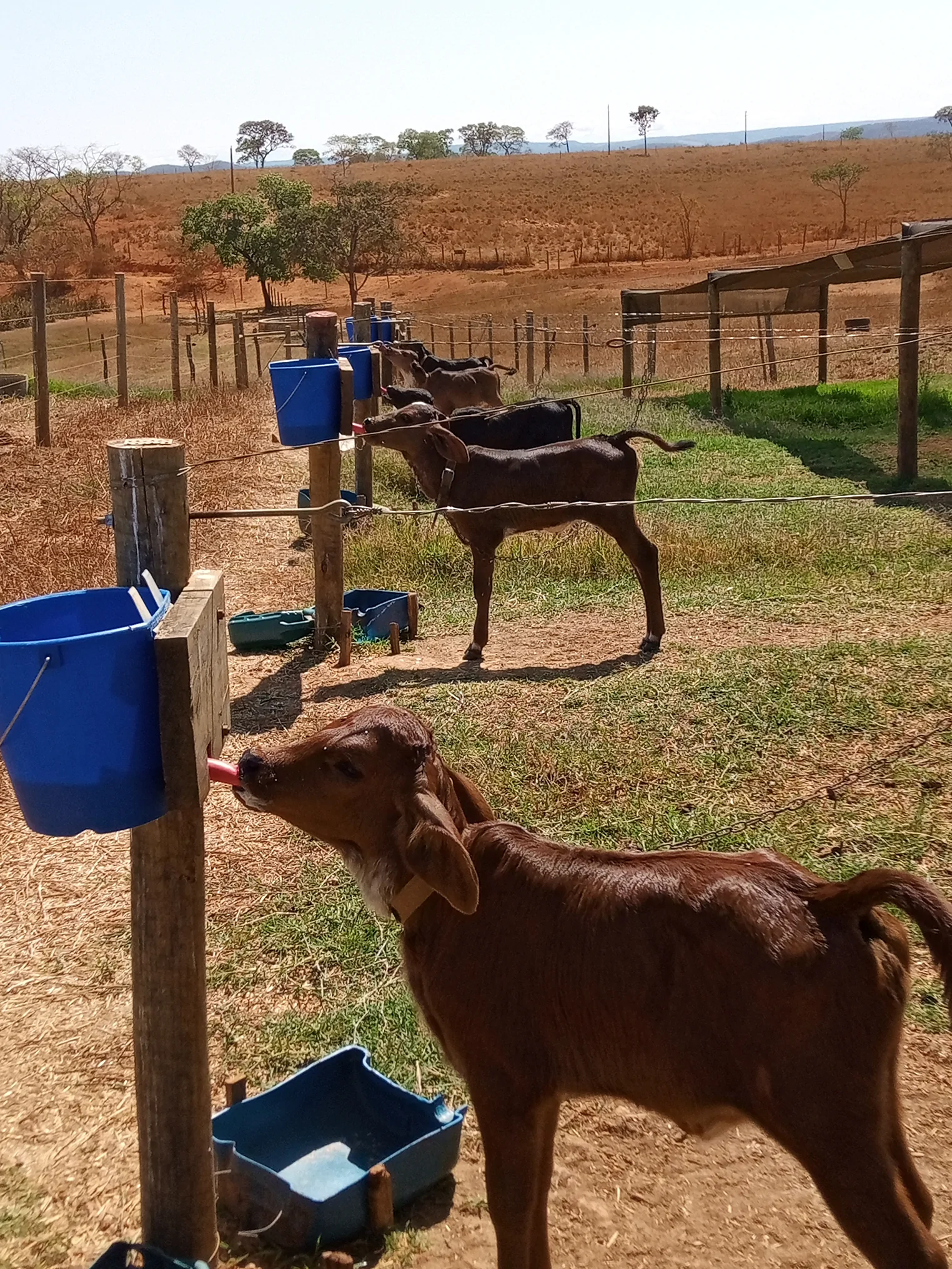
Calves housed in the tropical calf system

All procedures involving animals were approved by the Animal Care and Use Committee of the Universidade Federal de Minas Gerais, Brazil (protocol no. 73/2022). Twenty crossbred Gir x Holstein heifer calves (initial body weight 30.2 ± 3.47 kg) were enrolled. Calves were housed in a tropical calf-rearing facility equipped with individual buckets for milk, water, concentrate, and forage supply (Fig. 2).

Immediately after birth, calves were weighed and body measurements recorded. Doramectin was administered subcutaneously (200 µg/kg BW), and umbilical antisepsis was performed twice daily using 2.25% iodine solution until healing.

### Colostrum management and health procedures

Within the first hours of life, calves received high-quality colostrum (25 ± 1.96% Brix). Subsequently, 6 L/day of transition milk were provided until the third day of life, divided into two meals. A colostrum bank was available when necessary.

Passive transfer of immunity was evaluated by serum total protein concentration using a Brix refractometer. Blood samples were collected 48 h after the first colostrum feeding. Mean serum values (9.28 ± 0.98% Brix) indicated adequate passive transfer (Lombard et al. 2020; Shafiq et al. 2021). On day 3 of life, all calves received toltrazuril (20 mg/kg BW) as an anticoccidial treatment.

### Experimental design and treatment allocation

**Fig. 3:**
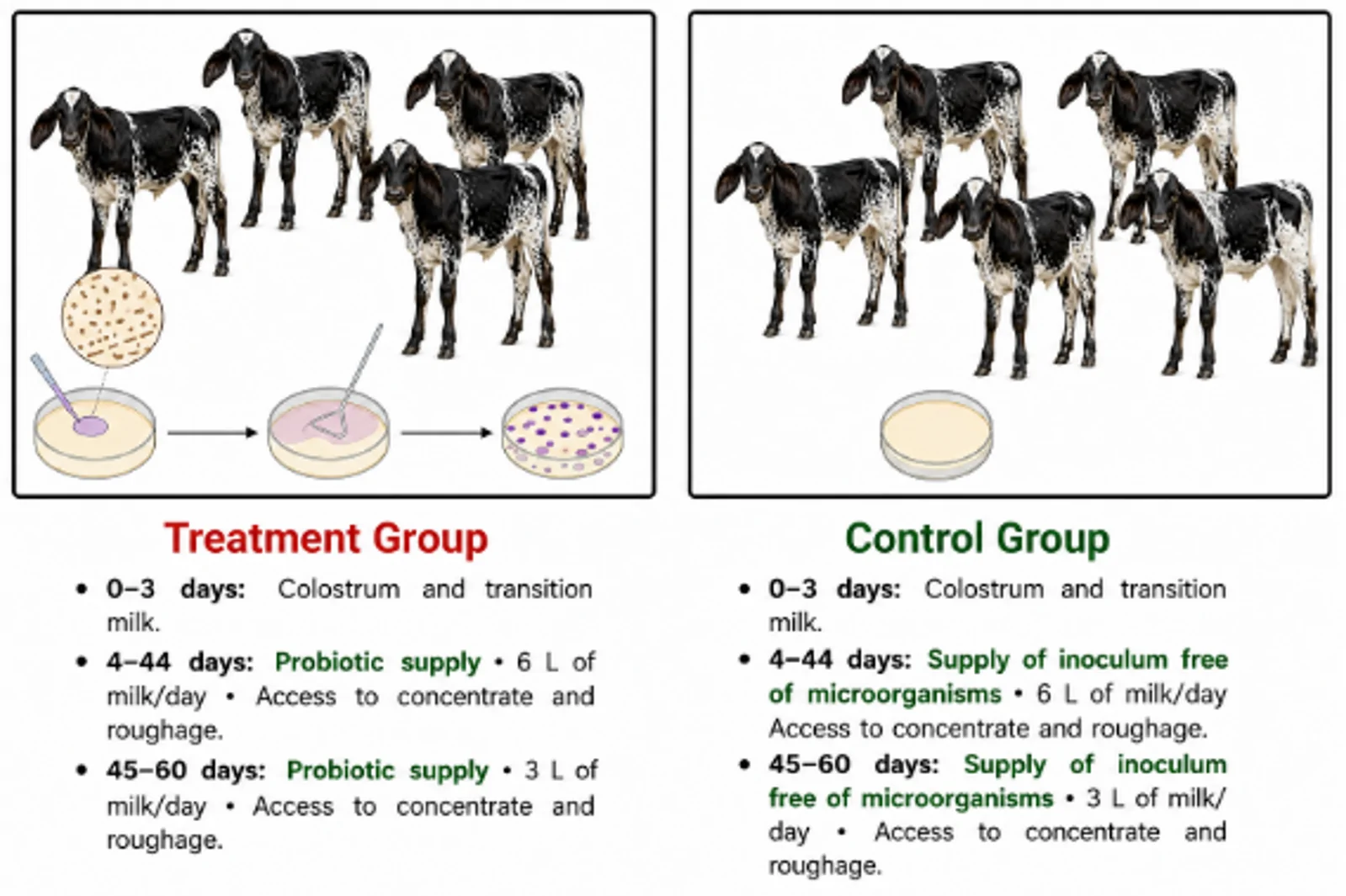
Experimental groups of calves and management procedures performed during each evaluation period

During each period, calves were assigned to either control or fungal supplementation treatments (*n* = 5 per treatment per period). Allocation was performed by random assignment of the first calf, followed by paired distribution according to birth order to maintain balanced groups. A total of 10 calves were evaluated per period (Fig. 3), totaling 20 animals.

### Feeding management and fungal supplementation

Calves were fed 6 L/day of milk divided into two meals until 44 days of age and 3 L/day once daily from 45 to 60 days. Water, starter concentrate, and elephant grass hay (*Pennisetum purpureum*) were provided ad libitum starting on day 4. The starter concentrate consisted of ground corn, soybean meal, dried distillers grains, corn gluten meal, and a mineral supplement (Table 1).

**Table 1:** Ingredient composition and chemical composition of diets fed to calves (g/kg dry matter basis)

Ingredient of conentrate			Inclusion rate (g/kg DM)		
Ground corn			515.5		
Soybean meal			103.1		
Dried distillers grains (DDG)			206.2		
Corn gluten meal			123.7		
Mineral supplement¹			51.5		
Chemical composition					
Item (g/kg DM)	Hay 1	Hay 2	Milk 1	Milk 2	Concentrate
Dry matter	923.4	901	116.6	124.9	886
Crude protein (CP)	44.6	78.1	46.4	35.7	199.5
Ether extract (EE)	22.7	34.9	21.5	14.4	45.2
Neutral detergent fiber (NDF)	631.3	675	-	-	187.2
Acid detergent fiber (ADF)	394.2	343	-	-	61.3
Mineral Matter	117.8	98.3	-	-	83.8

¹Mineral supplement composition (per kg of product): calcium, 255 g; cobalt, 48 mg; copper, 1,180 mg; sulfur, 9,800 mg; fluoride, 600 mg; phosphorus, 60 g; iodine, 55 mg; manganese, 1,480 mg; selenium, 15 mg; sodium, 120 g; zinc, 2,300 mg. Hay 1 = hay supplied during Period 1; Hay 2 = hay supplied during Period

Treated calves received 2 mL of Sabouraud broth containing 1 × 10⁸ CFU/mL of each microorganism administered orally once daily before milk feeding. Control calves received sterile broth only. Animals were monitored until weaning at 60 days of age.

### Feed intake measurement and pasture evaluation

Feed offered was adjusted to allow approximately 10% refusals. Orts were weighed daily to determine dry matter intake (DMI), and subsamples were stored for chemical analyses. Pasture intake was estimated using three 1-m² exclusion quadrats per period placed in *Cynodon nlemfuensis* paddocks. Forage mass was harvested at grazing height and weighed to estimate biomass production and intake.

### Chemical analyses

Feed, forage, concentrate, and ort samples were dried (55 ± 5 °C for 72 h), ground (1-mm screen), and analyzed according to AOAC (1998) procedures for dry matter, ash, crude protein, and ether extract. Neutral detergent fiber and acid detergent fiber were determined according to Van Soest et al. (1991) using an ANKOM fiber analyzer. Non-fiber carbohydrates were calculated as:

$$\mathrm{NFC}~(\%) = 100 - (\mathrm{NDF} + \mathrm{CP} + \mathrm{EE} + \mathrm{Ash})$$

Milk samples were analyzed for nitrogen content using the Kjeldahl method, and crude protein was estimated using a conversion factor of 6.38. Total solids were determined by the gravimetric method.

### Intake calculations and performance variables

Total DMI was calculated as the sum of milk, concentrate, and forage DMI. Feed conversion ratio (FCR) was calculated as: FCR = total DMI / total body weight gain.

Performance variables included: average daily gain (ADG), total weight gain, feed conversion ratio, nutrient intakes (CP, NDF, ADF, EE, and NFC). Body measurements and body weight were recorded at birth, day 4, every 15 days thereafter, and at weaning.

### Statistical analysis

Statistical analyses were performed using SAS (SAS Institute Inc., Cary, NC, USA), adopting a significance level of 0.05. Descriptive statistics were obtained using MEANS. Correlations were assessed using CORR, and outliers were identified using ROBUSTREG. Covariates (initial body weight, genetic composition, and parity order) were tested using GLMSELECT. Final measurements were analyzed by ANOVA using the GLM procedure. Repeated measurements were analyzed using the MIXED procedure, including animal as a random effect. For variables with multiple time points, random intercept and slope effects were fitted.

## Results

Initial body weight (IBW), genetic composition (GC), and parity order (PO) were analyzed as covariates. IBW significantly influenced hip width gain (HWG) and thoracic diameter gain (TDG) (*p* < 0.05). Parity order influenced withers height gain (WHG), whereas GC influenced the TDG (*p* < 0.05; Table 2).

**Table 2:** Mean total gain values (cm) and standard error of the mean (SEM) of body development measurements of Gir × Holstein treated or not with autochthonous fungi during two experimental periods

Variable	Period 1				Period 2				Significances covariates					
(cm)	Fungi	SEM	Control	SEM	Fungi	SEM	Control	SEM	T	*P*	T × *P*	Initial BW	Genetic group	Parity order
WHG	12.60	2.48	12.80	2.56	14.80	2.64	14.80	1.04	0.943	0.134	0.943	–	–	0.046
HHG	14.60	2.08	16.20	1.84	15.20	3.04	13.40	1.12	0.937	0.391	0.181	–	–	–
TDG	22.60	1.68	21.40	1.52	21.40	3.12	23.20	2.16	0.813	0.818	0.254	0.008	0.025	–
BLG	8.40	2.08	8.40	2.08	12.20	2.72	10.80	4.24	0.708	0.092	0.700	–	–	–

Notes: SEM = Standard error of the mean; T = Treatment; P = Period; T × P = Treatment × Period interaction. Covariates: Initial BW = Initial body weight; Genetic group = Genetic composition; Parity order = Dam parity. WHG= Withers height gain (cm); HHG = Hip height gain (cm); TDG = Thoracic diameter gain ; BLG =body length gain (cm) and – = not significant (*P* > 0.05)

**Fig. 4:**
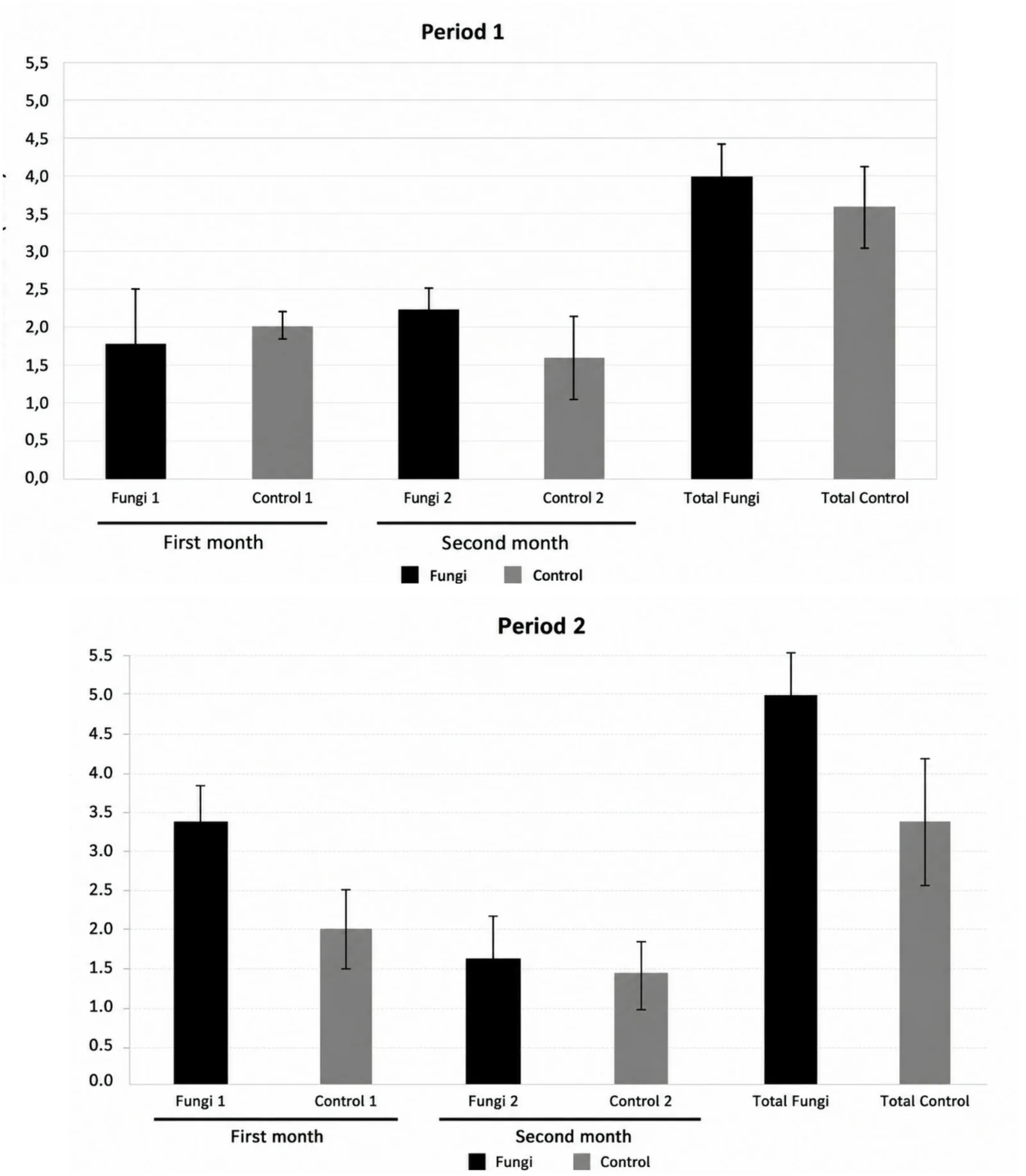
Mean monthly gain values (cm) and standard error of the mean (SEM) of hip width gain in Gir x Holstein, raised in tropical system, treated or not with fungi during two experimental period

**Fig. 5:**
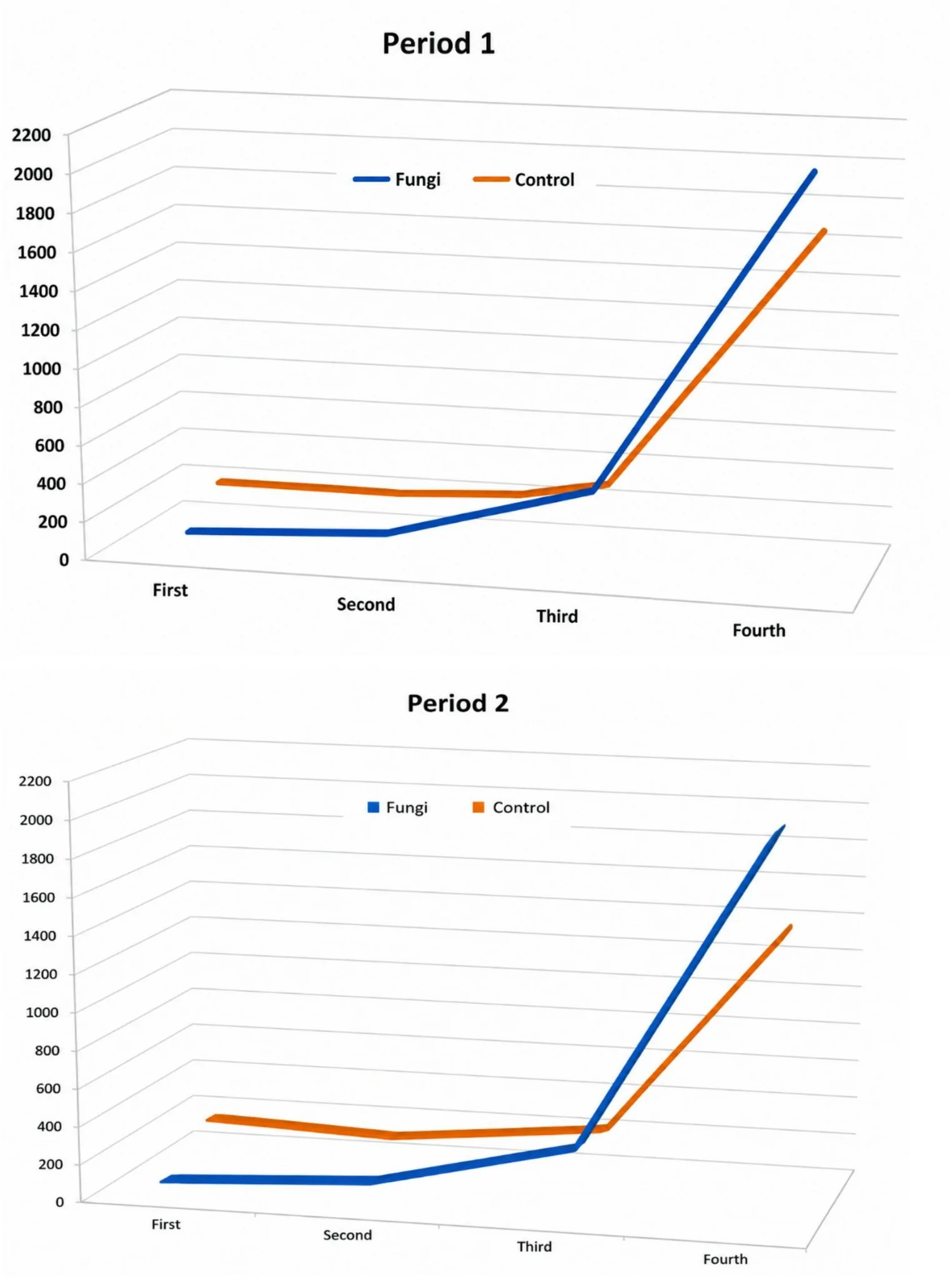
Biweekly intake of dry matter hay (gram) in Gir x Holstein, raised in tropical system, treated or not with fungi during two experimental period. Calves treated with the fungi tended to have greater hay DMI (*p* = 0.0640)

Mixed-model analysis showed a significant effect of calf age (*p* < 0.05) on concentrate and hay dry matter intake (DMI), as well as structural growth traits, including WHG, hip height gain (HHG), and TDG. Experimental period and fungal supplementation also influenced performance. Calves receiving fungi showed greater HWG compared with the control group in both periods (Suplementary Material S2; Fig. 4). Specifically, during the fourth fortnight, calves supplemented with fungi tended to have higher hay DMI (2.33 ± 1.78) to kg compared with the control group (1. 46 ± 0.91 kg) (*p* = 0.064; Fig. 5).

**Fig. 6:**
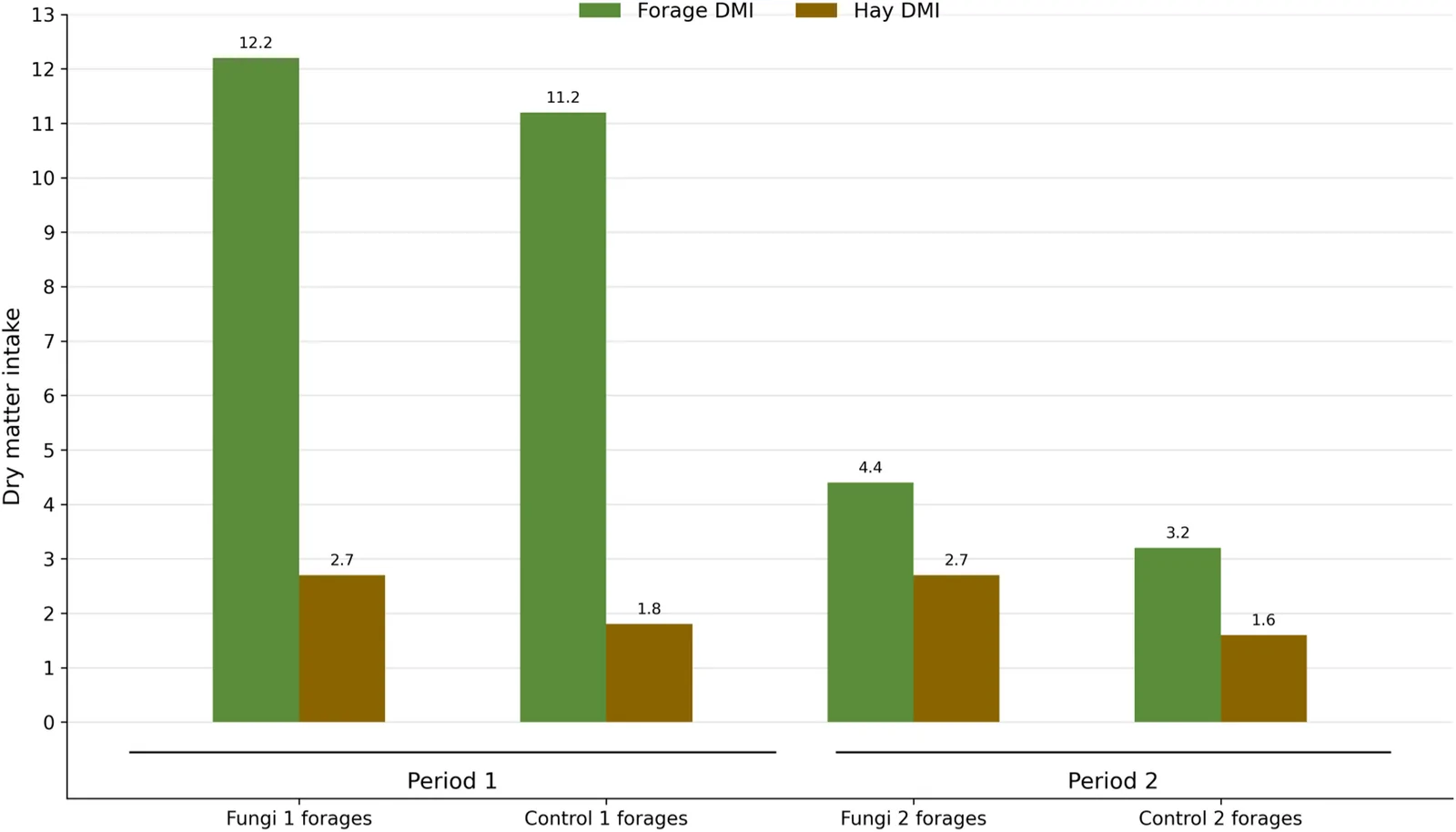
Averages (Kg) of total forage dry matter intake (DMI) and hay DMI in Gir x Holstein treated or not with fungi during two experimental periods raised in tropical system. The calves ingested a greater proportion of the forage in the calf barn compared to the hay offered

Fungal supplementation and the treatment × period interaction did not affect average daily gain (ADG) (*p* > 0.05). However, ADG was greater during period 2 (*p* < 0.01; Table 3). Concentrate DMI, hay DMI, total DMI, milk dry matter intake (MDMI), and feed efficiency (FE) were not influenced by treatment or the treatment × period interaction (*p* > 0.05). In contrast, concentrate DMI and FE were influenced by period, with improved values observed in period 2 (*p* < 0.01; Table 3). Covariates (IBW, GC, and PO) were not significant for these variables and were therefore removed from the final model. The concentrate: forage ratio was higher in Period 2 (*p* < 0.01; Table 2), however, it was not influenced by treatment or the interaction (TxP) (*p* > 0.01). The mean total forage DMI (hay and grass) during the 60 days of the study was 11.66 ± 2.01 and 3.75 ± 1.2 kg for Periods 1 and 2, respectively. The calves ingested a greater proportion of the forage present (*C. nlemfuensis*)compared to the hay offered in the calf barn (Fig. 6).

**Table 3:** Mean values and standard error of the mean (SEM) of growth performance, dry matter intake, and feed efficiency of Gi × Holstein crossbred heifers treated or not with autochthonous fungi during two experimental periods

Variable	Period 1				Period 2				Significances		
	Fungi	SEM	Control	SEM	Fungi	SEM	Control	SEM	T	*P*	T × *P*
Initial body weight (kg)	30.30	1.95	26.20	1.63	32.40	2.72	31.70	3.29	*	*	*
ADG (kg/day)	0.547	0.050	0.528	0.020	0.646	0.090	0.703	0.030	0.678	< 0.001	0.256
CI (g/day)	136.09	39.10	132.99	25.77	216.21	91.40	322.64	77.76	0.285	0.001	0.126
Hay intake (g/day)	44.86	28.78	31.92	11.80	45.99	38.00	26.60	15.10	0.250	0.882	0.826
C: F ratio	1.60	0.39	1.49	0.27	3,21	0.88	6.91	2,58	0.681	< 0.001	0.820
MDMI intake (kg)	35.06	0.67	34.05	1.08	38.22	0.15	38.07	0.18	0.548	< 0.001	0.335
DMI intake (g/day)	923.31	72.73	890.52	38.86	926.38	120.65	1010.91	73.44	0.583	0.190	0.204
Feed conversion ratio	1.70	0.10	1.69	0.05	1.44	0.06	1.44	0.10	0.966	< 0.001	0.881

Notes: SEM = standard error of the mean; ADG = average daily gain; DMI dry matter intake (g/day); CI = CI Concentrate dry matter intake (g/day), C:F = Concentrate: forage ratio; MDMI =Milk dry matter intake (kg), T = treatment; P = period; T × P = treatment × period interaction. * Not evaluated because initial body weight was analised as covariate

No effects of treatment, period, or their interaction were detected for daily DMI, crude protein (CPI), ether extract (EEI), neutral detergent fiber (NDFI), or non-fiber carbohydrates (NFCI) (*p* > 0.05; Table 4). However, acid detergent fiber intake (ADFI) was higher during period 1 (*p* < 0.01; Table 4).

**Table 4:** Mean values and standard error of the mean (SEM) for average daily nutrient intake (g/kg of DM) of crossbred Gir × Holstein heifers treated or not with autochthonous fungi during two experimental periods

Variables	Period 1 (winter–spring)				Period 2 (late summer–early autumn)				Significance (P-value)		
	Fungi	SEM	Control	SEM	Fungi	SEM	Control	SEM	Treatment	Period	T × P
DMI	923.31	40.09	890.52	20.51	899.22	65.31	983.75	39.39	0.5672	0.4465	0.2039
CP intake	76.06	5.24	74.20	3.04	78.39	12.66	93.22	7.82	0.4310	0.2024	0.3146
EE intake	77.34	1.89	74.82	1.56	71.13	2.64	75.25	1.82	0.6961	0.1711	0.1194
NDF intake	347.70	53.98	330.61	17.35	314.43	72.72	389.37	35.99	0.5671	0.8002	0.3664
ADF intake	74.13	8.17	60.93	5.52	37.78	6.11	37.84	3.43	0.3632	0.0006	0.3590
NFC intake	401.22	24.34	391.78	9.78	425.38	23.93	420.15	9.09	0.6844	0.1749	0.8992

Abbreviations: SEM = Standard error of the mean; DMI = Dry matter intake; CP = Crude protein; EE = Ether extract; NDF = Neutral detergent fiber; ADF = Acid detergent fiber; NFC = Non-fiber carbohydrates; Treatment = Fungal supplementation; Period = Experimental period; T × P = Treatment × Period interaction

## Discussion

The tropical calf hutch system (Argentine system) is widely adopted in Brazil and Argentina due to its low operating cost, suitability for tropical conditions, and potential to stimulate immune development through gradual exposure to environmental pathogens (Nunes et al. 2024; Oliveira et al. 2022). However, environmental factors such as ambient temperature and rainfall may influence calf performance and welfare, requiring appropriate housing design and management strategies. These factors may also affect the accurate measurement of feed intake, feed efficiency (FE), and growth parameters.

Most studies conducted under outdoor rearing systems have primarily focused on health and behavioral aspects (Campos et al. 2023), whereas information regarding growth performance, nutrient intake, and structural development of calves raised under these conditions—particularly across different seasons—remains limited. Therefore, the present study contributes to addressing this gap by evaluating productive and morphometric parameters under practical tropical conditions.

Structural growth was mainly influenced by intrinsic animal factors in the covariate analyses. Although no significant treatment × period interaction was observed for hip height gain (HHG) (*p* > 0.05), a numerical inversion between periods was noted. While this pattern was not statistically significant, it may reflect variability related to seasonal conditions or individual animal responses and should therefore be interpreted cautiously, without overestimating its biological relevance. In addition, birth weight affected hip width gain (HWG) and thoracic diameter gain (TDG), indicating that heavier calves at birth have greater early structural growth potential.

The greater TDG observed in calves with a higher Holstein genetic composition may be associated with increased body capacity and metabolic potential typical of this genetic group, including greater cardiopulmonary and digestive capacity (Panetto et al. 2020). The effect of parity on withers height gain (WHG) supports the association between multiparous cows and greater birth weights (Oliveira and Nogueira 2006), likely reflecting improved uterine development and enhanced fetal nutrient supply. A noticeable numerical difference between initial body weight (*p* > 0.05) between treatment groups was observed in Period 1. This variation was statistically controlled using covariate analysis. The observed difference may be related to the calving sequence, which determined group allocation (control vs. treated), as described in the Materials and Methods section.

Fungal supplementation increased HWG in both experimental periods. This trait is relevant for dairy cattle conformation, as it is associated with pelvic structure, calving ease, and mammary support (Carvalho 2018; Silva 2011). Genetic correlations also indicate a positive association between hip width and rear udder width (Esteves et al. 2004). Although future productive performance was not evaluated, these findings suggest a potential long-term structural effect that warrants further investigation.

Fungal supplementation did not affect average daily gain (ADG). One possible explanation is that calves received a nutritionally adequate diet (milk, starter concentrate, and forage), which supported proper rumen development and overall performance, thereby limiting the potential for additional probiotic effects.

Moreover, calves were managed under adequate sanitary conditions, likely resulting in low microbial challenge. Evidence suggests that probiotic effects tend to be more pronounced under conditions of stress or intestinal dysbiosis (Carroll et al. 2025). In the present study, calves doubled their birth weight by 60 days of age, indicating adequate performance regardless of supplementation.

Similar results were reported by Santos et al. (2021), who also observed no significant effect of fungal supplementation on ADG. However, Neto et al. (2014) reported increased ADG when bacterial probiotics and yeast were combined and supplementation extended into the post-weaning phase. In that study, *Lactobacillus casei* and *Bifidobacterium bifidum* were administered during the early milk-feeding phase, followed by *Saccharomyces cerevisiae* and *Pediococcus acidilactici* during weaning, suggesting that responses may depend on microbial combinations and supplementation protocols.

The greater ADG observed during late summer–early autumn may be associated with improved forage quality and greater concentrate dry matter intake (DMI) during this phase. Increased concentrate intake promotes short-chain fatty acid (SCFA) production, particularly propionate and butyrate, which stimulate rumen epithelial development and enhance absorptive capacity (Scholz et al. 2011; Oliveira et al. 2019).

Additionally, the improved FE in Period 2, despite similar total DMI, supports the hypothesis of enhanced nutrient utilization efficiency, possibly related to improved diet quality and a higher concentrate-to-forage ratio. Although fungal strains have demonstrated the ability to produce fibrolytic enzymes in in vitro studies (Duarte et al. 2021, 2025), this potential was apparently insufficient to alter fiber digestion in vivo or increase total dry matter intake during the preweaning phase. However, more pronounced effects might occur after the complete establishment of ruminal functionality, typically between the third and fourth month of life, which should be analyzed in future studies.

Dry matter intake of calves raised in individual outdoor systems has been poorly documented under tropical conditions. However, total dry matter intake for calves under eight weeks of age ranges from 1.17 to 3.06% of body weight (National Academies of Sciences, 2021). Thus, the values observed in this study were within the expected range. Concentrate and hay intake increased with age, as expected during the transition from pre-ruminant to functional ruminant (Torrezan et al. 2016). The gradual reduction in milk supply at 45 days of age stimulated solid feed intake.

The relatively low hay intake can be explained by the availability of fresh forage in the outdoor system, suggesting a preference for green pasture. Indeed, forage dry matter intake (grass) was quantitatively higher than hay intake, indicating that calves actively selected fresh forage when available. This behavior is consistent with the natural feeding pattern of young ruminants, which preferentially consume more palatable and digestible feeds, particularly those with higher moisture content and nutritional quality.

Feed efficiency was not influenced by supplementation, corroborating previous studies evaluating probiotics in calves and small ruminants (Júnior et al. 2022; Magaço et al. 2020; Meyer et al. 2001). Responses to microbial additives depend on multiple factors, including environmental stress, diet quality, and forage-to-concentrate ratio (Magaço et al. 2020).

Overall, supplementation with autochthonous fungi exerted a specific effect on structural development (HWG) but did not influence ADG, DMI, or FE during the preweaning period. The experimental period had a more consistent impact on growth performance and feed efficiency than microbial supplementation. Howeve, long-term studies evaluating reproductive performance, milk yield, and ruminal parameters are required to determine whether the observed structural effects translate into future productive advantages.

## Conclusion

Supplementation with *Trichoderma longibrachiatum* and *Rhodotorula mucilaginosa* isolates derived from the bovine gastrointestinal tract increased monthly hip width gain in Gir × Holstein dairy calves. Hay dry matter intake tended to be greater during the fourth fortnight in supplemented calves.

Overall performance, including average daily gain (ADG), concentrate and forage dry matter intake (DMI), and feed efficiency (FE), was greater during the late summer–early autumn period compared with the late winter–spring period when calves were reared under an outdoor hutch system in a semi-arid tropical region.

## Supplementary Information

Below is the link to the electronic supplementary material.

Supplementary Material 1

Supplementary Material 2

## Data Availability

The data that support the findings of this study are available on request from the corresponding author.
