# Supplementary material for: Growth performance and nutrient intake of Gir x Holstein dairy calves reared in a tropical outdoor system and inoculated with rumen-derived fungi
Source: Trop Anim Health Prod. 2026 Jul 21;58(7):440. doi: 10.1007/s11250-026-05248-7 (PMC13388535; doi:10.1007/s11250-026-05248-7)
Supplement: Supplementary file 1 — Supplementary Material 1 [file 11250_2026_5248_MOESM1_ESM.doc]

# Growth performance and nutrient intake of Gir × Holstein dairy calves reared in a tropical outdoor system and inoculated with rumen-derived fungi

# Ellen Batista Pereira^a^, Luciana Castro Gerassev^a^, Felipe Gomes da Silva^a^, Leila Magalhães Queiróz^a^, Flávio Emanuel Gomes Silva^a^, Júlia de Melo Viana^a^, Idael Matheus Góes Lopes^a^,Eduardo Robson Duarte^a*^

^*^Corresponding author: [duartevet@hotmail.com](mailto:duartevet@hotmail.com)

**Suplementar Material 1**:. _Average monthly temperatures and relative air humidity during two experimental periods


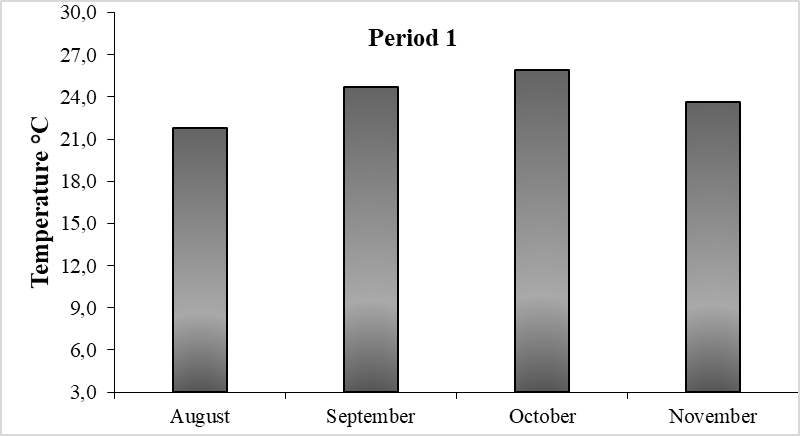

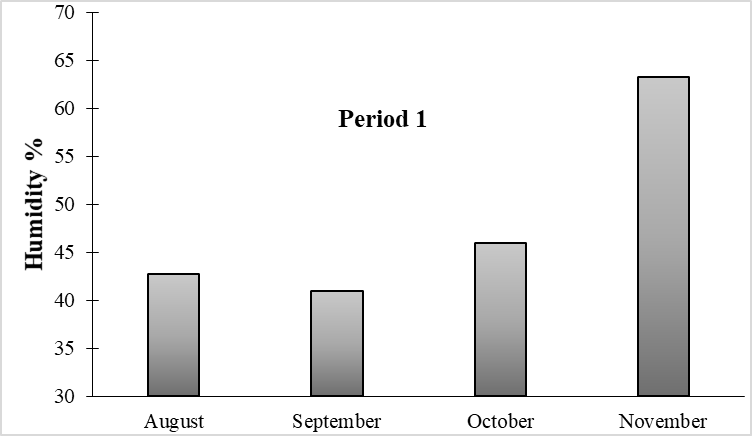

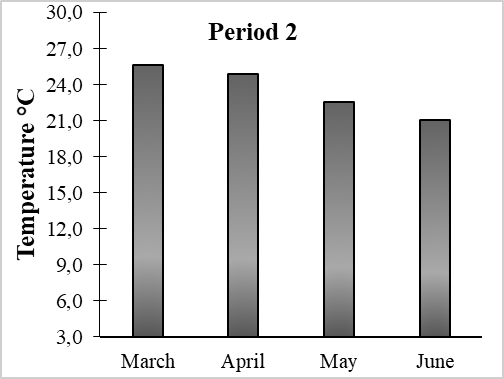

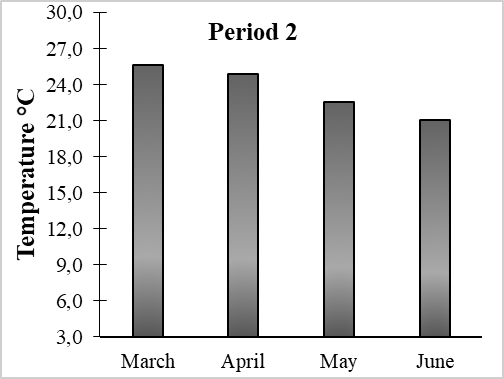


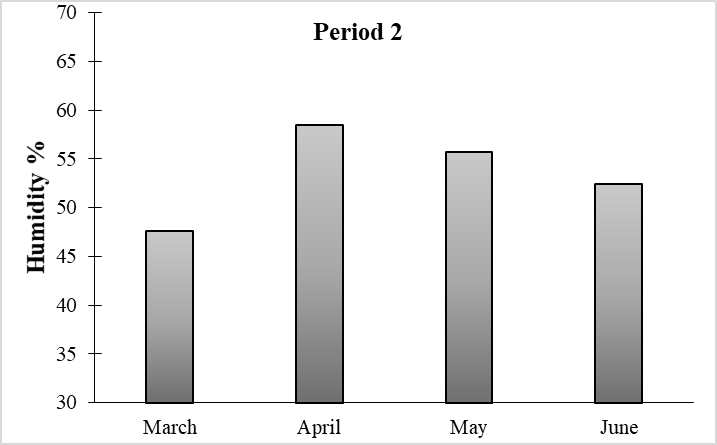


**Idade dos animais**
