# Supplementary material for: Growth performance and nutrient intake of Gir x Holstein dairy calves reared in a tropical outdoor system and inoculated with rumen-derived fungi
Source: Trop Anim Health Prod. 2026 Jul 21;58(7):440. doi: 10.1007/s11250-026-05248-7 (PMC13388535; doi:10.1007/s11250-026-05248-7)
Supplement: Supplementary file 2 — Supplementary Material 2 [file 11250_2026_5248_MOESM2_ESM.docx]

# Growth performance and nutrient intake of Gir × Holstein dairy calves reared in a tropical outdoor system and inoculated with rumen-derived fungi

# Ellen Batista Pereira^a^, Luciana Castro Gerassev^a^, Felipe Gomes da Silva^a^, Leila Magalhães Queiróz^a^, Flávio Emanuel Gomes Silva^a^, Júlia de Melo Viana^a^, Idael Matheus Góes Lopes^a^,Eduardo Robson Duarte^a*^

^*^Corresponding author: [duartevet@hotmail.com](mailto:duartevet@hotmail.com)

**Supplementary Material S2- Table .** P-values for variables related to weight gain, hay and concentrate intake, and body development of Holstein × Gir heifers treated or not with autochthonous fungi across two experimental periods and according to age.

| Variables | | Treatment | Period | T × P | Age |
| --- | --- | --- | --- | --- | --- |
| Fortnightly analyses | n |  |  |  |  |
| ADG | 80 | 0.6352 | 0.0007 | 0.3685 | 0.3799 |
| Concentrate DMI | 80 | 0.7188 | 0.0092 | 0.6107 | 0.0001 |
| Hay DMI | 80 | 0.2537 | 0.0690 | 0.7878 | 0.0001 |
| Monthly analyses | |  |  |  |  |
| Variable | n | Treatment | Period | T × P | Age |
| Withers height gain | 40 | 0.8505 | 0.1179 | 0.7744 | 0.0048 |
| Hip height gain | 40 | 0.6507 | 0.1173 | 0.1637 | 0.0023 |
| Hip width gain | 40 | 0.0491 | 0.0025 | 0.7845 | 0.2224 |
| Thoracic diameter gain | 40 | 0.9982 | 0.8703 | 0.2393 | 0.0010 |
| Body length gain | 40 | 0.9756 | 0.7589 | 0.9916 | 0.5678 |

Abbreviations: n = number of observations; Treatment = fungal supplementation; Period = experimental period; T × P = treatment × period interaction; Age = effect of calf age; ADG = average daily gain; DMI = dry matter intake.
